# Comparative Effects of Heart Failure Medications on Cardiac Remodeling via the Hydrogen Sulfide (H_2_S) Pathway: A Systematic Review

**DOI:** 10.3390/diseases14080299

**Published:** 2026-08-18

**Authors:** Mohamed Thabit Ahmed, Bashir A. Yousef, Gonen Ozsarlak-Sozer, Tahir Yagdi, Sanem Nalbantgil, Emine Nur Ozbek, Elmoiz Babekir, Khalid A. Ateyyah, Mohammed Ahmed Zahrani, Sultan Almuallem, Alaa Mousli, Mohammed Basendowah

**Affiliations:** 1 Department of Pharmacology, Faculty of Pharmacy, Omdurman Islamic University, Khartoum 14415, Sudan; 2 Department of Pharmacology, Faculty of Pharmacy, Ege University, Izmir 35100, Turkey; 3 Department of Clinical Pharmacy and Pharmacology, Ibn Sina National College of Medical Studies, Jeddah 22421, Saudi Arabia; 4 Department of Pharmacology, Faculty of Pharmacy, University of Khartoum, Khartoum 11111, Sudan; 5 Department of Cardiovascular Surgery, Faculty of Medicine, Ege University, Izmir 35100, Turkey; 6 Department of Cardiology, Faculty of Medicine, Ege University, Izmir 35100, Turkey; 7 Department of Medicine and Adult Critical Care Medicine, Ibn Sina College for Medical Studies, Jeddah 22421, Saudi Arabia; 8 College of Medicine, Taibah University, Madinah 42353, Saudi Arabia; 9 Department of Medicine, Faculty of Medicine, King Abdulaziz University, Jeddah 21589, Saudi Arabia; 10 Department of Surgery, Faculty of Medicine, King Abdulaziz University, Jeddah 21589, Saudi Arabia

**Keywords:** heart failure, hydrogen sulfide, cardiac remodeling, cytoprotective, pharmacotherapy

## Abstract

Objectives: The aim of this study was to determine whether direct evidence shows that contemporary guideline-directed heart failure (HF) pharmacotherapies influence cardiac remodeling through hydrogen sulfide (H_2_S) signaling, and to define the resulting evidence gap. Design: A systematic review was conducted according to PRISMA 2020. Registration: PROSPERO CRD420251238589. Data Sources: MEDLINE (PubMed), Web of Science, Scopus, and the Cochrane Library; searches were initiated in March 2025, covered publications from January 2007 onward, and were last updated in November 2025. Eligibility Criteria: Primary studies had to include (A) an HF or cardiac-remodeling model, (B) an HF-relevant pharmacological intervention, (C) direct assessment or manipulation of H_2_S biology, and (D) at least one cardiac-remodeling endpoint. Results: Of the 40,796 unique records screened, 50 reports underwent full-text assessment and 14 unique studies were included. No study demonstrated that the remodeling benefits of ACE inhibitors, ARBs, ARNIs, beta-blockers, MRAs, hydralazine/isosorbide dinitrate, or SGLT2 inhibitors are mediated by endogenous H_2_S signaling. Doiron et al. evaluated empagliflozin with or without the H_2_S donor SG1002 in experimental HFpEF; the combination improved several outcomes beyond empagliflozin alone, but this adjunctive design does not establish H_2_S mediation of empagliflozin action. Most eligible evidence concerned exogenous H_2_S donors in animal models and reported improvements in fibrosis, hypertrophy, oxidative stress, mitochondrial injury, and cardiac function. The overall certainty was low because of preclinical predominance, heterogeneous models and H_2_S assays, donor-specific pharmacology, and incompletely reported randomization and blinding. Conclusions: The principal finding is negative but clinically important: direct evidence that H_2_S mediates established HF pharmacotherapy is currently absent. H_2_S remains a promising experimental therapeutic candidate rather than an established shared mechanism or clinically validated treatment target.

## 1. Introduction

Heart failure (HF) is driven by progressive cardiac remodeling—a maladaptive process involving ventricular hypertrophy, chamber dilation, extracellular matrix expansion, mitochondrial dysfunction, and myocyte loss that ultimately impairs pump performance and worsens clinical outcomes [1,2]. Preventing or reversing this structural deterioration remains a central objective of HF management [2]. Guideline-directed pharmacotherapies, including renin–angiotensin–aldosterone system (RAAS) inhibitors, β-blockers, mineralocorticoid receptor antagonists (MRAs), angiotensin receptor–neprilysin inhibitors (ARNIs), and sodium–glucose cotransporter 2 (SGLT2) inhibitors, are proven to improve ventricular function and promote reverse remodeling across diverse HF phenotypes [3,4,5,6,7]. However, the mechanistic basis for these structural benefits remains incompletely understood.

Hydrogen sulfide (H_2_S) has emerged as a key endogenous gasotransmitter with broad cytoprotective actions in the cardiovascular system. Circulating and myocardial H_2_S levels are frequently reduced in HF, and lower concentrations correlate with more advanced disease [8]. Experimental evidence demonstrates that H_2_S mitigates oxidative stress, apoptosis, inflammation, mitochondrial injury, and fibrosis, core drivers of adverse remodeling [9,10,11]. Restoring H_2_S signaling via exogenous donors or by enhancing endogenous biosynthesis (CSE, CBS, 3-MST) consistently improves cardiac structure and function in preclinical HF models [12,13]. RAAS activation and β-adrenergic overstimulation, central features of HF, suppress H_2_S biosynthesis [14,15], whereas pathway blockade may restore H_2_S availability, suggesting potential mechanistic convergence.

### 1.1. Rationale and Knowledge Gap

Despite extensive study of individual HF drug classes, the possibility that multiple guideline-directed therapies converge mechanistically on the H_2_S pathway has not been systematically examined. Because H_2_S integrates redox homeostasis, inflammation, mitochondrial integrity, and extracellular matrix remodeling, it has been proposed as a possible point of mechanistic convergence; however, this remains a hypothesis. No prior review has mapped the direct integrated evidence, indirect mechanistic evidence, and evidence gaps linking major HF drug classes, H_2_S biology, and cardiac remodeling across clinical and preclinical studies.

**Figure 1:**
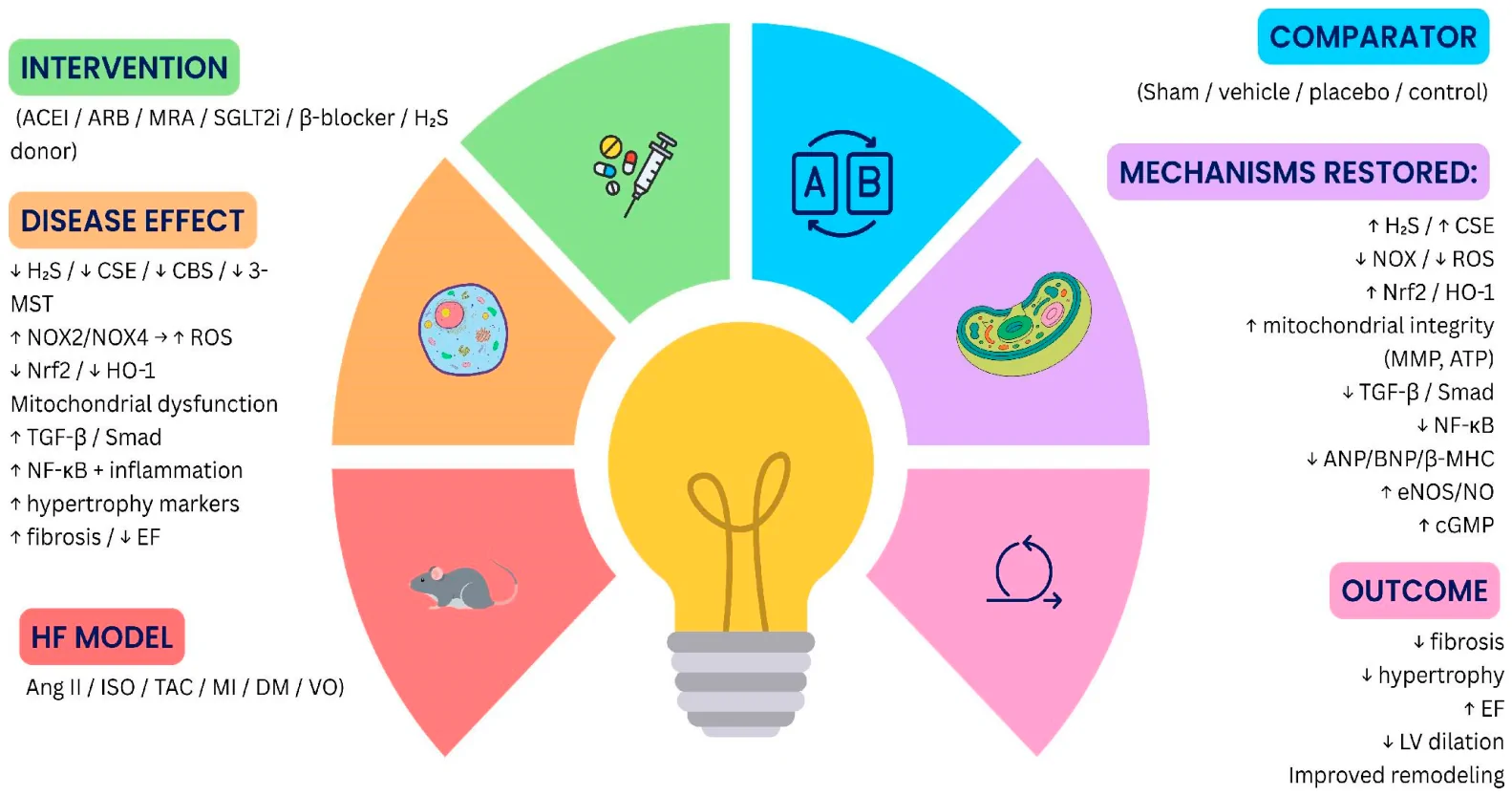
Conceptual framework for HF drug mechanisms.

To provide a structured approach for interpreting heterogeneous animal models, pharmacological interventions, and H_2_S-related outcomes, we developed a conceptual framework linking: (1) HF models, (2) disease-induced alterations in H_2_S signaling, (3) drug classes evaluated in the literature, (4) comparators, (5) mechanisms restored by treatment, and (6) downstream cardiac remodeling outcomes. The framework was used to distinguish directly evaluated relationships from indirect or hypothesis-generating links and guided the data extraction strategy, outcome grouping, and synthesis throughout the review (Figure 1).

### 1.2. Objectives

This systematic review evaluates whether direct evidence supports the hypothesis that guideline-directed HF medications influence cardiac remodeling through modulation of H_2_S signaling. Specifically, we assess the following:

- Effects of major HF drug classes (ACE inhibitors, ARBs, beta-blockers, MRAs, ARNI, and SGLT2 inhibitors) on H_2_S production and biosynthetic enzymes (CSE, CBS, and 3-MST), where these outcomes were directly measured.
- Whether improvements in remodeling outcomes (fibrosis, hypertrophy, and oxidative injury) were directly associated with H_2_S-related changes within the same experimental design.
- The consistency and limitations of direct, indirect, preclinical, and clinical evidence.
- Whether current evidence demonstrates, or only supports as a hypothesis, that H_2_S represents a shared downstream mediator across HF pharmacotherapies.

By synthesizing evidence from 2007 to 2025, this review tests rather than assumes a unifying mechanistic pathway and identifies the studies required to establish or refute H_2_S-mediated effects of contemporary HF therapies.

## 2. Materials and Methods

### 2.1. Protocol Registration

This systematic review was conducted in accordance with PRISMA 2020 (Preferred Reporting Items for Systematic Reviews and Meta-Analyses), the updated reporting guideline for systematic reviews and meta-analyses that includes a 27-item checklist and flow-diagram templates to improve transparent reporting [16]. The review protocol was prospectively registered in PROSPERO (the International Prospective Register of Systematic Reviews), a public registry that promotes protocol transparency and helps reduce avoidable duplication and selective reporting, under registration number CRD420251238589. The full protocol (including eligibility criteria, search strategy, and planned synthesis methods) was finalized and made publicly available before data extraction commenced.

### 2.2. Eligibility Criteria

Eligibility criteria were established to determine the appropriateness of studies for inclusion in the review. Eligibility was structured using a PICOS (Population, Intervention, Comparator, Outcomes, and Study) design. PICO denotes the first four elements and is commonly used to frame intervention questions, whereas PICOS adds study design to make eligibility boundaries explicit (Table 1).

Study designs and populations: We included primary research studies of any design that examined the effects of heart-failure (HF) pharmacotherapies on indices of cardiac remodeling and assessed H_2_S levels or H_2_S-related mechanisms. Eligible designs comprised:

- Randomized or non-randomized clinical trials in patients with HF;
- Observational clinical studies (prospective or retrospective cohorts, registries, and case–control studies) enrolling HF populations;
- Preclinical in vivo HF models (e.g., post–myocardial infarction remodeling, pressure-overload cardiomyopathy, volume overload, metabolic cardiomyopathy);
- In vitro cardiac or myocardial cell models linked explicitly to HF or post-injury remodeling.

In line with contemporary ACC/AHA and ESC guidance, HF was considered a clinical syndrome characterized by typical symptoms and/or signs with objective evidence of cardiac dysfunction or elevated filling pressures [17,18]. For preclinical work, we required models to demonstrate features compatible with HF-level remodeling (e.g., impaired systolic or diastolic function, chamber dilation, elevated filling pressures, or progressive post-infarction remodeling), rather than isolated hypertrophy alone.

Conceptual distinction between hypertrophy, remodeling, and HF. Because the terms cardiac hypertrophy, ventricular remodeling, and heart failure are often used interchangeably, we prespecified a framework to avoid misclassification. Cardiac hypertrophy was defined as an increase in myocardial mass and/or cardiomyocyte size in response to hemodynamic or molecular stimuli, which may be physiological or pathological but does not necessarily constitute clinical HF if global pump function and filling pressures remain preserved [2,19]. Ventricular remodeling was defined as the broader structural and geometric changes (altered chamber size, mass, wall thickness, and fibrosis) that develop after cardiac injury or chronic neurohormonal activation and are closely linked to HF progression [19,20]. Accordingly, isolated hypertrophy models (e.g., early TAC or Ang II without clear remodeling) and purely mechanistic hypertrophy studies were classified as hypertrophy-focused mechanistic models. Because our synthesis requires a demonstrable cardiac-remodeling phenotype, these studies were excluded from the primary HF + H_2_S synthesis, while their H_2_S-related insights were retained for mechanistic discussion.

Interventions: We focused on guideline-directed HF pharmacotherapies and H_2_S-targeted approaches. Eligible interventions included:

- Renin–angiotensin–aldosterone system (RAAS) inhibitors (ACE inhibitors and ARBs);
- Angiotensin receptor–neprilysin inhibitor (ARNI);
- β-adrenergic blockers;
- Mineralocorticoid receptor antagonists (MRAs);
- Sodium–glucose cotransporter-2 (SGLT2) inhibitors;
- Hydralazine-based regimens (e.g., hydralazine with isosorbide dinitrate);
- Direct or slow-releasing H_2_S donors and H_2_S-releasing hybrids, either alone or in combination with standard HF therapies.

Combination regimens (e.g., RAAS blockade plus an H_2_S donor, or hydralazine/isosorbide dinitrate in HF) were eligible if they reported both remodeling and H_2_S-related outcomes.

Comparators: We accepted placebo or no-treatment controls, active comparators (e.g., another HF drug), and baseline or sham-operated groups, according to the underlying study design. For animal and cell experiments, eligible studies compared intervention versus control conditions with respect to both remodeling and H_2_S-pathway indices.

Outcomes and core HF+H_2_S criteria: To be included in the primary HF + H_2_S synthesis, studies had to satisfy all the following four core criteria:

- A defined HF model or HF patient population beyond isolated hypertrophy;
- Administration of an HF-relevant pharmacological intervention (guideline-directed therapy or H_2_S-targeted agent);
- Assessment of H_2_S biology (measured H_2_S levels; expression or activity of H_2_S-producing enzymes such as CSE, CBS, or 3-MST; or downstream pathways known to be modulated by H_2_S);
- Reporting at least one cardiac remodeling outcome, such as left ventricular ejection fraction or volumes, indices of systolic or diastolic function, infarct size, fibrosis area, hypertrophy markers, or remodeling-related biomarkers (e.g., natriuretic peptides, collagen content).

Studies that contributed only to H_2_S biology without a clear HF model, or that reported HF remodeling outcomes without any H_2_S-pathway assessment, were excluded from the primary dataset but could still inform the conceptual and mechanistic discussion.

Language and publication status: No restrictions were placed on publication status. Non-English papers were considered if an English abstract or a reliable translation was available.

Finally, all eligible studies were assigned to one of several primary synthesis groups according to their predominant HF drug class (RAAS blockade, β-blockers, MRAs, ARNI, SGLT2 inhibitors, hydralazine/ISDN, and direct H_2_S-targeted therapies).

### 2.3. Search Strategy

The search strategy was designed a priori in line with PRISMA 2020 guidance [16]. We constructed three conceptual blocks: (i) heart failure and ventricular remodeling; (ii) H_2_S biology; and (iii) HF pharmacotherapies of interest. For the HF/remodeling block, we used a combination of Medical Subject Headings (MeSH) and free-text terms for “heart failure”, “ventricular remodeling”, “left ventricular hypertrophy”, “myocardial fibrosis” and related constructs, informed by contemporary epidemiological and pathophysiological literature and the 2023 ESC HF guideline [2,17,18,19,20,21]. The H_2_S block included terms such as “hydrogen sulfide”, “H_2_S”, “cystathionine gamma-lyase”, “cystathionine β-synthase”, and “3-mercaptopyruvate sulfurtransferase” [22,23,24]. The pharmacotherapy block covered drug classes and representative molecules (e.g., “ACE inhibitor”, “ARB”, “sacubitril/valsartan”, “spironolactone”, “eplerenone”, “carvedilol”, “metoprolol”, “dapagliflozin”, “empagliflozin”, “hydralazine”, “isosorbide dinitrate”, “GYY4137”, “S propargyl-cysteine”, “thiosulfate”, “SG1002”).

We searched MEDLINE (via PubMed), Web of Science Core Collection, Scopus, and the Cochrane Library. The review process began following PROSPERO registration in 2025; database coverage extended from 1 January 2007 to the final search update conducted prior to manuscript submission. The 2007 start date was chosen to capture the emergence of cardiovascular H_2_S research. Search strategies combined three mandatory concept blocks—HF/remodeling, H_2_S biology, and HF pharmacotherapy—with database-specific controlled vocabulary, free-text terms, field restrictions, and Boolean operators. Complete reproducible strings are reported in Table 2. No language filter was applied at the search stage.

### 2.4. Study Selection

**Figure 2:**
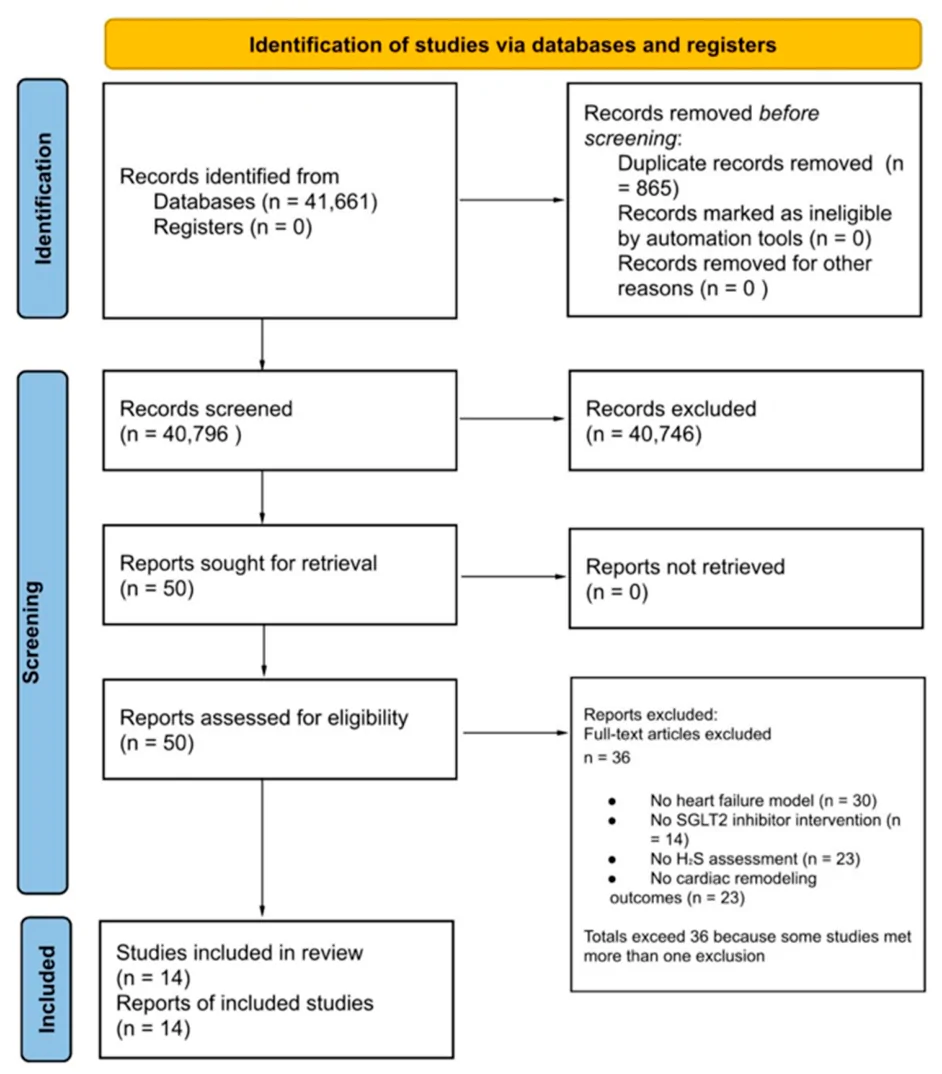
PRISMA 2020 flow diagram of study selection.

The database searches yielded 41,661 records. After removal of 865 duplicates, 40,796 unique records underwent title and abstract screening. Of these, 40,746 were excluded, leaving 50 reports for full-text assessment. Thirty-six full-text reports were excluded and 14 unique studies were included in the qualitative synthesis (Figure 2). The arithmetic is therefore internally consistent: 40,796 screened − 40,746 excluded = 50 full texts, and 50 − 36 = 14 included studies.

Duplicate citations were identified before screening by comparing DOI/PMID and bibliographic fields (title, first author, year, journal), followed by manual review of possible near-duplicates. Screening decisions were documented against the predefined eligibility criteria. Full-text eligibility and extracted study characteristics were cross-checked within the review team using a standardized spreadsheet, and disagreements were resolved by discussion and consensus. No formal inter-reviewer agreement coefficient was calculated.

Of these, 36 studies were excluded at the full-text stage. The principal reasons for exclusion were absence of a heart failure model or HF patients (Criterion A; 30 studies), absence of an HF-related pharmacological intervention (Criterion B; 14 studies), lack of H_2_S assessments or H_2_S–related enzyme measurements (Criterion C; 23 studies), and absence of cardiac remodeling endpoints (Criterion D; 23 studies). Several studies failed more than one criterion, and therefore, category totals exceed 36. A total of 14 studies met all inclusion criteria and were included in the qualitative synthesis. The full PRISMA 2020 flow diagram is presented in Figure 2.

### 2.5. Data Items

For each study, we mapped extracted outcomes onto a predefined conceptual framework linking HF models, interventions, disease effects, mechanisms restored, comparators, and primary cardiac remodeling endpoints. Remodeling outcomes included left ventricular ejection fraction, ventricular volumes, infarct size, fibrosis area, hypertrophy indices, and natriuretic peptides. H_2_S-pathway outcomes included plasma or tissue H_2_S, CSE/CBS/3-MST expression or activity, and downstream mediators such as Nrf2 signaling, oxidative stress markers, mitochondrial function, and inflammatory pathways. Where possible, we distinguished between acute and chronic intervention windows.

For interpretation, studies were stratified by clinical proximity into overt HF/cardiomyopathy models and remodeling-surrogate models. This prevents isolated hypertrophy or fibrosis experiments from being treated as equivalent to chronic human HF and allows conclusions to be weighted according to model relevance.

### 2.6. Risk of Bias Assessment

Risk of bias (RoB) was assessed using design-appropriate tools. Randomized clinical trials were evaluated with the revised Cochrane RoB 2 tool, non-randomized human studies with ROBINS-I, and animal experiments with the SYRCLE risk-of-bias tool [25,26,27]. Three reviewers independently rated each domain, with disagreements resolved by consensus or consultation with a third reviewer. In addition, we implemented a semi-automated Google Apps Script pipeline linked to our extraction spreadsheet, which acted as a “machine reviewer” to flag potentially high- or unclear-risk domains based on predefined logical rules (e.g., missing statements on randomization, blinding, or allocation concealment). These automated flags were used only to support reviewer judgment; all final RoB assessments remained human decisions. Detailed domain-level SYRCLE risk-of-bias ratings are presented in Table 3. We did not exclude studies solely based on RoB, but RoB judgements were used to qualify the strength and certainty of the evidence in the narrative synthesis.

### 2.7. Data Synthesis

Studies were grouped according to their primary intervention class (RAAS blockade, β-blockers, MRAs, ARNI, SGLT2 inhibitors, hydralazine/ISDN, and direct H_2_S donors). Given the substantial heterogeneity in study designs, HF phenotypes, dosing regimens, comparators, and outcome reporting, a quantitative meta-analysis was not feasible. We therefore conducted a qualitative narrative synthesis. Within each drug-class group, we performed the following:

- Summarized the effects of the intervention on cardiac remodeling outcomes;
- Synthesized evidence on H_2_S-pathway modulation (levels, enzymes, and downstream signaling);
- Noted consistencies and discrepancies across studies, including differences between preclinical and clinical evidence;
- Highlighted dose–response patterns and differences between acute and chronic treatment where data allowed.

Mechanistic and hypertrophy-only studies that did not meet the full HF + H_2_S inclusion criteria were not part of the primary dataset but were integrated into the discussion to contextualize and interpret the HF findings. For interpretation, direct integrated evidence was defined as a study assessing an HF or remodeling model, a pharmacological intervention, H_2_S biology, and remodeling outcomes within the same design. Evidence addressing only some of these elements was classified as indirect mechanistic evidence, and proposed links not directly tested were identified as hypothesis-generating. No statistical transformations or imputations were performed; data were synthesized as reported. Because no quantitative meta-analyses were undertaken, we did not conduct formal subgroup analyses, meta-regression, or GRADE assessments, and the certainty of evidence remains qualitatively inferred. Results of individual studies and syntheses are presented in structured tables and accompanying narrative text.

### 2.8. Data Collection Process

Two reviewers independently extracted data from all eligible studies using a standardized data extraction form. Extracted information included study characteristics, intervention details, heart failure model, H_2_S-related outcomes, cardiac remodeling outcomes, and key mechanistic findings. Any disagreements were resolved through discussion and, when necessary, consultation with a third reviewer. The study authors were not contacted for additional or missing data. Automation tools were not used for data extraction; however, a Google Apps Script was later used to assist the organization and review of extracted data.

## 3. Results

### 3.1. Study Selection and Characteristics

The literature search and screening process yielded 14 unique studies after correction of the duplicate Ahmad 2016/Ahmad 2016b entry. The final set comprised nine overt HF or cardiomyopathy models and five remodeling-surrogate models. These categories are reported separately in Table 4 and are not treated as clinically equivalent.

The included evidence was predominantly preclinical and heterogeneous. Overt HF/cardiomyopathy models included post-infarction, pressure-overload, volume-overload, catecholamine-associated, diabetic, toxic, cardiometabolic HFpEF, and cardiorenal HF models. Remodeling surrogates included isolated or early pressure-overload hypertrophy, angiotensin II-associated hypertensive remodeling, and hypertension-related collagen remodeling. Favorable results across these models indicate biological activity of H_2_S-targeted interventions but do not establish efficacy in chronic human HF or mediation of GDMT effects.

Importantly, one eligible preclinical cardiometabolic HFpEF study evaluated empagliflozin alone and in combination with the H_2_S donor SG1002 while reporting H_2_S-related and remodeling outcomes [31]. This study supports potential additivity of H_2_S supplementation but does not establish that empagliflozin increases H_2_S or that its remodeling effects are H_2_S-mediated. No comparable integrated study was identified for ACE inhibitors, ARBs, ARNIs, beta-blockers, MRAs, or hydralazine/ISDN. By contrast, a subset of preclinical H_2_S-donor studies in HF or HF-related remodeling models satisfied the full HF + H_2_S + remodeling criteria and formed the core mechanistic dataset for this review.

Several additional studies partially addressed individual components of our framework. For example, large clinical HF trials of MRAs and SGLT2 inhibitors reported remodeling or hard outcomes but did not assess H_2_S or its enzymes [3,4,5,6,7,38,39]; mechanistic hybrid HFpEF work with SGLT2 inhibition quantified detailed diastolic and redox endpoints without interrogating H_2_S [40]; and several mechanistic studies measured H_2_S in the context of HF-related stressors or HF drugs but without a full HF-remodeling framework [41,42,43,44,45]. These studies were therefore excluded from the core HF + H_2_S + remodeling dataset but are used in the discussion to provide a mechanistic context.

Risk of bias was predominantly unclear rather than demonstrably low. Most animal studies did not report sufficient information on sequence generation, allocation concealment, random housing, or blinding. Accordingly, the certainty of the preclinical evidence was judged low to very low and the apparent consistency of favorable donor effects should not be interpreted as high-certainty efficacy. Domain-level judgments are shown in Table 3 and were explicitly incorporated into the interpretation.

The findings of the included studies are organized below by major classes of HF medication and by direct H_2_S donor therapy. The narrative emphasizes both remodeling outcomes (improvements or worsening of cardiac structure/function) and H_2_S-related findings (changes in H_2_S levels or signaling pathways) for each therapy class, and, where possible, compares clinical or translational data with preclinical models to assess whether mechanistic insights align.

Across the major drug classes, the central result was the absence of evidence demonstrating that established HF pharmacotherapies exert their remodeling effects through H_2_S signaling. The Doiron et al. preclinical combination study is an important exception to the general absence of integrated designs, but it supports potential additivity rather than mediation. Purpose-designed preclinical and clinical studies are required to test whether standard HF drugs modulate cardiac remodeling through H_2_S signaling (Table 5).

### 3.2. RAAS Inhibitors (ACE Inhibitors, ARBs, ARNI) and the H_2_S Pathway

No preclinical or clinical HF study was identified in which ACE inhibitors, ARBs, or ARNI therapy were tested in an HF population or model with simultaneous assessment of (i) H_2_S levels or H_2_S-producing enzymes and (ii) quantitative cardiac remodeling outcomes. Instead, the evidence base for RAAS blockers and H_2_S consists of (i) HF-or HF-like models in which H_2_S donors counteracted Ang II–driven remodeling [28,29,30,34,46]; and (ii) mechanistic studies where thiol-containing ACEIs modified H_2_S/NO biology in ischemia–reperfusion or vascular-injury settings rather than in established HF [41,42,43].

Across multiple preclinical studies, activation of RAAS has been consistently associated with suppression of endogenous H_2_S signaling and downregulation of cystathionine-γ-lyase (CSE), the principal enzymatic source of cardiovascular H_2_S [28,34,47,48,49]. Ang II infusion reduces myocardial CSE expression and lowers cardiac and plasma H_2_S levels, leading to oxidative stress, hypertrophy, mitochondrial injury, and fibrosis [28,48,49].

Pharmacological RAAS blockade with either ACE inhibitors or ARBs restores CSE expression and normalizes myocardial H_2_S levels, attenuating hypertrophy and collagen deposition [28,30]. Additional mechanistic evidence demonstrates that H_2_S directly counteracts pathways activated by Ang II. In isoproterenol (ISO)-induced hypertrophy, exogenous H_2_S (via NaHS) suppresses mitochondrial NOX4, reduces ROS generation, prevents cytochrome c release, preserves mitochondrial membrane potential, and decreases caspase-3 activation [15]. These mechanisms closely parallel those observed in Ang II–induced remodeling, in which NOX-dependent oxidative stress and mitochondrial dysfunction act as major drivers of hypertrophy and fibrosis.

Pressure-overload (transverse aortic constriction, TAC) models also demonstrate marked depression of endogenous H_2_S during pathological hypertrophy [29]. H_2_S donor therapy restores myocardial H_2_S levels, activates PI3K/Akt–Nrf2 antioxidant defense pathways, and reverses hypertrophic remodeling [29,46]. Thiol-containing ACE inhibitors such as zofenopril and captopril provide further indirect support for this interaction. Zofenopril increases circulating H_2_S and NO bioavailability and confers superior cardio-protection compared with non-thiol ACEIs, attributable to thiol-mediated H_2_S release and synergistic H_2_S–NO signaling [41,43,50].

Although direct evidence for interactions between ARNI therapy and H_2_S signaling remains limited, neprilysin inhibition increases natriuretic peptide (NP) activity and augments cGMP and NO pathways that exhibit mechanistic overlap with H_2_S. RAAS overactivation can suppress the myocardial CSE/H_2_S system, whereas separate experimental studies report restoration of H_2_S-related pathways under RAAS blockade. These observations provide biological plausibility only; they do not demonstrate that H_2_S mediates the clinical reverse-remodeling effects of ACEIs, ARB, or ARNI therapy.

### 3.3. β-Blockers and the H_2_S Pathway

Similarly, no clinical or preclinical HF study was found in which β-blocker therapy was evaluated in an HF population or model with concurrent measurement of H_2_S and detailed remodeling endpoints according to our full criteria. Available data instead come from: (i) mechanistic dosing studies of carvedilol in healthy mice that quantified H_2_S in the absence of HF remodeling [44]; (ii) a chronic canine ischemic cardiomyopathy model in which carvedilol and metoprolol improved function and metabolic remodeling without direct assessment of H_2_S [45]; and (iii) preclinical H_2_S-donor studies in catecholamine-induced HF models.

Across preclinical models of chronic beta-adrenergic stimulation, excessive catecholamine activity is associated with reduced endogenous H_2_S and disruption of cytoprotective pathways. Separate non-HF or mechanistic studies suggest that beta-blockers, particularly carvedilol, may influence myocardial H_2_S-related measures. Because no integrated HF + beta-blocker + H_2_S + remodeling study was identified, this relationship remains hypothesis-generating rather than an established component of beta-blocker action.

Multiple independent studies have shown that isoproterenol administration reduces intracellular and cardiac H_2_S and induces hypertrophy, oxidative stress, and mitochondrial injury [15]. Supplementation with H_2_S prevented these effects by activating glucose-6-phosphate dehydrogenase (G6PD), increasing NADPH availability, lowering the NADP+/NADPH ratio, downregulating p53, and restoring redox homeostasis [15]. In vivo, NaHS maintained sarcomeric structure, preserved mitochondrial membrane potential, reduced cytochrome c release, and inhibited caspase-3 activation in ISO- and TAC-induced heart failure [15,29].

Direct modulation of H_2_S by beta-blockers has been documented mainly in non-HF settings. In SJL mice, carvedilol administered intraperitoneally for five consecutive days (10 mg/kg) increased myocardial H_2_S concentrations by approximately 75% compared with control animals [44]. A recent metabolomics study in a chronic canine HF model reported that beta-blocker therapy improved LV function and altered sulfur-containing and mitochondrial metabolites without directly demonstrating H_2_S-mediated remodeling [45]. Together, these observations support a possible NO-H_2_S-mitochondrial hypothesis, but integrated evidence remains lacking.

### 3.4. Mineralocorticoid Receptor Antagonists (MRAs) and the H_2_S Pathway

Large, randomized trials have established that MRAs such as spironolactone and eplerenone improve survival and induce reverse remodeling in HFrEF [3,4,38]. However, none of these pivotal studies measured H_2_S levels, CSE/CBS/3-MST expression, or other explicit H_2_S-pathway markers, and no preclinical HF model combining MRAs with formal H_2_S assessment met all predefined criteria. Consequently, the link between MRAs and H_2_S signaling in HF remains inferential and is reconstructed from separate lines of evidence rather than from integrated HF + H_2_S studies.

Excess aldosterone contributes to heart failure progression by promoting myocardial fibrosis and by increasing oxidative stress and inflammation [51]. MRAs reduce these processes and improve LV structure and function in HF, but none of the included clinical studies measured H_2_S before and after MRA therapy, and preclinical data are sparse. Similarities between MRA effects and pathways modified by H_2_S donors are indirect and do not establish that MRAs preserve or act through endogenous H_2_S signaling. Dedicated HF + MRA + H_2_S remodeling studies are required.

### 3.5. SGLT2 Inhibitors and the H_2_S Pathway

SGLT2 inhibitors have become a cornerstone in contemporary heart failure management across the LVEF spectrum, with robust reductions in HF hospitalization and cardiovascular death in HFrEF and HFmrEF/HFpEF [5,6,7,39]. None of these landmark trials, however, reported measurements of H_2_S, H_2_S-synthesising enzymes, or explicit H_2_S-pathway endpoints. Mechanistic hybrid work in human and experimental HFpEF myocardium demonstrated that empagliflozin can partially normalize microvascular inflammation, oxidative stress, and passive cardiomyocyte stiffness [40], but again without interrogating H_2_S signaling directly.

A recent preclinical study by Doiron et al. provides the only direct link between SGLT2 inhibition and the H_2_S pathway in cardiometabolic HFpEF [52]. In two independent HFpEF models driven by metabolic stress and hypertension, animals were randomized to SGLT2 inhibitor monotherapy, oral H_2_S donor (SG1002) alone, or the combination. While SGLT2 inhibition improved diastolic indices and exercise capacity, co-administration of SG1002 produced substantially higher circulating H_2_S levels and yielded superior improvements in diastolic function (lower E/e′), exercise tolerance, and interstitial fibrosis in both heart and kidney compared with SGLT2 inhibition alone [52]. Notably, SG1002 monotherapy also improved diastolic function and histological markers, consistent with its previously reported ability to increase H_2_S and nitric oxide bioavailability in HF patients [53].

From a mechanistic perspective, SGLT2 inhibitors have not yet been shown to directly upregulate CSE/CBS/3-MST or to increase endogenous H_2_S production. The Doiron study showed that adding SG1002 to empagliflozin increased H_2_S-related measures and produced greater improvements than empagliflozin alone [31]. This supports potential adjunctive benefit of H_2_S supplementation, but it does not demonstrate that empagliflozin acts through H_2_S. Integrated human HF studies remain absent.

### 3.6. Other Therapies and Considerations: Hydralazine/ISDN and Direct H_2_S Donors

Hydralazine plus isosorbide dinitrate (ISDN) is an established vasodilator regimen for heart failure, particularly in patients who cannot tolerate ACEI/ARB therapy or in specific populations (e.g., African American patients with HFrEF). The main benefit of hydralazine/ISDN is hemodynamic: hydralazine reduces afterload through arterial dilatation, while ISDN reduces preload via vasodilatation. In our review, we did not identify any preclinical or clinical studies that directly measured H_2_S levels, CSE expression, or downstream H_2_S signaling in the context of hydralazine or hydralazine/ISDN therapy.

No direct evidence was identified concerning hydralazine/ISDN and H_2_S signaling. The absence of evidence does not justify classifying the regimen as biologically neutral; it means that no mechanistic conclusion can presently be drawn.

## 4. Discussion

This systematic review demonstrates a major evidence gap: no study establishes that H_2_S signaling mediates the remodeling benefits of guideline-directed HF therapy. The two relevant bodies of evidence must remain separate. Established HF drugs improve clinical and remodeling outcomes, generally without measuring H_2_S; conversely, direct H_2_S-targeted interventions improve remodeling-related endpoints mainly in experimental models. Their coexistence supports a testable hypothesis, not mechanistic convergence or mediation.

Doiron et al. evaluated empagliflozin alone, SG1002 alone, and their combination in two experimental HFpEF models [31]. Greater benefit with the combination supports possible adjunctive or additive effects of exogenous H_2_S augmentation. It does not demonstrate that empagliflozin increases endogenous H_2_S, that H_2_S lies downstream of SGLT2 inhibition, or that empagliflozin requires H_2_S signaling to improve remodeling.

Across GDMT classes, several indirect mechanistic links emerge. RAAS activation suppresses CSE/H_2_S and promotes oxidative, fibrotic, and mitochondrial injury [2,19,20,28,49,54], while H_2_S donors reverse several of these processes [28,29,30,34,46]. Sulfhydryl ACEIs such as zofenopril and captopril can influence H_2_S and NO bioavailability in non-HF or ischemia–reperfusion settings [41,42,43,50]. These observations support biological plausibility but do not show that H_2_S mediates reverse remodeling during RAAS inhibition in HF.

Adrenergic stress reduces H_2_S availability during hypertrophy and metabolic injury [14,15], and H_2_S donors can restore redox balance and mitochondrial function [15,29]. Carvedilol has altered myocardial H_2_S-related measures in non-HF models [44], but clinical beta-blocker trials did not measure H_2_S. Likewise, MRAs and H_2_S donors affect overlapping oxidative and fibrotic pathways, yet no integrated study demonstrates an H_2_S-mediated component of MRA benefit. These relationships are hypothesis-generating.

SGLT2 inhibitors improve HF outcomes [5,6,7,39] and modulate inflammation, stiffness, and oxidative stress [40]. The Doiron study suggests that exogenous H_2_S augmentation may provide additional benefit beyond SGLT2 inhibition [31], but it does not establish a shared downstream mechanism. Accordingly, H_2_S should currently be regarded as an experimental therapeutic candidate and a testable mechanistic hypothesis, not as a central or established mediator of contemporary HF pharmacotherapy.

### 4.1. Study Limitations

The evidence must be interpreted conservatively. First, it is overwhelmingly preclinical. The included experiments span pressure overload, isoproterenol or angiotensin II exposure, post-infarction or volume-overload HF, diabetic cardiomyopathy, doxorubicin cardiotoxicity, and cardiometabolic HFpEF. These models reproduce selected components of human HF and were therefore stratified as overt HF/cardiomyopathy models or remodeling surrogates rather than treated as clinically equivalent. Second, donor pharmacology is heterogeneous. Rapid sulfide salts such as NaHS generate brief concentration peaks that may not reproduce endogenous enzymatic signaling, whereas thiosulfate, DATS, SG1002, S-propargyl-cysteine, and hybrid H_2_S-NO compounds have different release kinetics, metabolites, tissue distribution, and potential off-target actions. Third, H_2_S measurement lacks standardization: free sulfide, acid-labile or total sulfide, sulfane sulfur, enzyme expression/activity, and downstream signaling markers are related but non-interchangeable. Pre-analytical oxidation, sample handling, and assay choice further limit cross-study comparison. Fourth, many animal reports did not adequately describe sequence generation, allocation concealment, random housing, or blinding; the resulting certainty is low to very low despite generally consistent favorable donor signals. Fifth, clinical translation remains minimal. SG1002 has limited early-phase human data concerning H_2_S/NO bioavailability, but no convincing clinical trial has shown durable reverse remodeling, fewer HF hospitalizations, improved symptoms, or lower mortality through H_2_S augmentation. Finally, publication bias and selective outcome reporting cannot be excluded, and no meta-analysis or formal GRADE assessment was feasible. Broader mechanistic literature on H_2_S–NO/redox crosstalk and CSE regulation [55,56,57,58,59], and major HF pharmacotherapy outcome trials [60,61,62,63], is acknowledged here as background context; it was not used to support causal claims within this review, consistent with the evidence-gap framing above.

### 4.2. Strengths and Overall Contribution

Despite these limitations, this review has several notable strengths. It systematically maps mechanistic, preclinical, and clinical data across major HF therapies using explicit HF + H_2_S + remodeling criteria. This approach identifies which studies directly interrogate the proposed axis and shows transparently that direct evidence for H_2_S mediation of guideline-directed therapy is largely absent. The side-by-side comparison separates established remodeling effects of HF drugs, experimental effects of H_2_S donors, and indirect mechanistic observations. The resulting framework is therefore evidence-gap oriented and hypothesis-generating rather than proof of a shared cytoprotective pathway.

### 4.3. Clinical Implications

The present findings do not support a change in clinical HF treatment. H_2_S-guided drug selection, routine H_2_S biomarker testing, or adjunctive donor therapy cannot be recommended outside research. The appropriate implication is experimental: purpose-designed studies should test whether standard HF drugs alter validated H_2_S measures, whether pharmacological or genetic interruption of H_2_S signaling attenuates drug benefit, and whether carefully characterized donors add clinically meaningful benefit to contemporary GDMT.

### 4.4. Future Directions

To translate these mechanistic insights into clinical practice, future work should prioritize: prospective HF cohorts in which H_2_S and its metabolites are measured longitudinally alongside echocardiographic and biomarker indices of remodeling under guideline-directed therapy; randomized phase II trials evaluating adjunctive H_2_S donors (or H_2_S-enhancing compounds) on top of RAAS blockade, β-blockade, MRAs, ARNI, or SGLT2 inhibitors, with predefined remodeling endpoints; development and validation of standardized analytical platforms for H_2_S quantification (including sulphane sulphur and enzyme expression) to enable cross-study harmonization; mechanistic studies dissecting whether MRAs, ARNIs, and SGLT2 inhibitors directly modulate H_2_S biosynthesis (CSE/CBS/3-MST) or primarily preserve H_2_S signaling by lowering oxidative and inflammatory burden; integrative work linking microvascular dysfunction, ECM remodeling, and mitochondrial injury to specific nodes within the CSE/H_2_S axis in human HF tissue.

## 5. Conclusions

This systematic review identifies a substantial evidence gap rather than a proven unifying mechanism. Current studies do not demonstrate that H_2_S mediates the remodeling benefits of ACE inhibitors, ARBs, ARNIs, beta-blockers, MRAs, SGLT2 inhibitors, or hydralazine/isosorbide dinitrate. The Doiron study supports possible additivity between empagliflozin and exogenous SG1002 in experimental HFpEF, but it does not establish that empagliflozin acts through H_2_S. Exogenous H_2_S donors repeatedly improve remodeling-related outcomes in animal models, supporting H_2_S as a biologically plausible experimental therapeutic candidate. However, model heterogeneity, donor pharmacokinetics, non-standardized H_2_S measurements, unclear risk-of-bias domains, and limited human evidence preclude causal or clinical claims. Purpose-designed mechanistic studies and adequately powered clinical trials are required before H_2_S can be regarded as a mediator, biomarker, or therapeutic target in routine HF care.

## Figures and Tables

**Table 1:** PICOS Framework for Eligibility Criteria.

PICOS Component	Eligibility Criterion
Population	Adult animal models or translational models of heart failure or left ventricular hypertrophy (LVH).
Intervention	Pharmacological agents directly or indirectly modulating hydrogen sulfide (H_2_S) pathways or established heart-failure drug classes.
Comparator	Placebo, no treatment, or standard-therapy control arms.
Outcomes	Cardiac remodelling endpoints (LV mass, hypertrophy indices, fibrosis, collagen deposition), biochemical H_2_S levels, and mechanistic pathway biomarkers.
Study Design	Randomised or controlled preclinical studies; translational mechanistic investigations adhering to predefined methodological and reporting standards.

Abbreviations: H_2_S, hydrogen sulfide; LVH, left ventricular hypertrophy; PICOS, Population, Intervention, Comparator, Outcomes, and Study design.

**Table 2:** Database coverage, search timing, and conceptual structure.

Database and Timing	Fields/Vocabulary	Search Structure and Adaptation
MEDLINE (PubMed) Coverage: January 2007–October 2025 Initial search: following PROSPERO registration (2026).	MeSH plus title/abstract fields	HF/remodeling AND H_2_S biology AND HF drug classes/donor terms; no language filter at the search stage.
Web of Science Core Collection Coverage: January 2007–October 2025 Initial search: following PROSPERO registration (2026).	Topic fields; keyword vocabulary	The same three mandatory concept blocks; controlled vocabulary is not available.
Scopus Coverage: January 2007–October 2025 Initial search: following PROSPERO registration (2026).	TITLE-ABS-KEY	The same three mandatory concept blocks; automatic synonym expansion disabled.
Cochrane Library Coverage: January 2007–October 2025 Initial search: following PROSPERO registration (2026).	MeSH and title/abstract/keyword fields	HF/remodeling AND H_2_S AND drug/donor terms; no trial-status filter.

Abbreviations: ACEI, angiotensin-converting enzyme inhibitor; ARB, angiotensin receptor blocker; ARNI, angiotensin receptor-neprilysin inhibitor; H_2_S, hydrogen sulfide; HF, heart failure; MeSH, Medical Subject Headings; PRISMA, Preferred Reporting Items for Systematic Reviews and Meta-Analyses; SGLT2, sodium–glucose cotransporter 2. Complete reproducible search strings: MEDLINE (PubMed): ((“Heart Failure”[Mesh] OR “heart failure”[tiab] OR “ventricular remodeling”[tiab] OR “cardiac remodeling”[tiab] OR “left ventricular hypertrophy”[tiab] OR “myocardial fibrosis”[tiab]) AND (“Hydrogen Sulfide”[Mesh] OR “hydrogen sulfide”[tiab] OR H_2_S[tiab] OR “cystathionine gamma-lyase”[tiab] OR CSE[tiab] OR “cystathionine beta-synthase”[tiab] OR CBS[tiab] OR “3-mercaptopyruvate sulfurtransferase”[tiab] OR 3-MST[tiab]) AND (“Angiotensin-Converting Enzyme Inhibitors”[Mesh] OR “Angiotensin Receptor Antagonists”[Mesh] OR “Adrenergic beta-Antagonists”[Mesh] OR “Mineralocorticoid Receptor Antagonists”[Mesh] OR “Sodium-Glucose Transporter 2 Inhibitors”[Mesh] OR ACE inhibitor*[tiab] OR ARB[tiab] OR sacubitril[tiab] OR valsartan[tiab] OR spironolactone[tiab] OR eplerenone[tiab] OR carvedilol[tiab] OR metoprolol[tiab] OR dapagliflozin[tiab] OR empagliflozin[tiab] OR hydralazine[tiab] OR “isosorbide dinitrate”[tiab] OR GYY4137[tiab] OR NaHS[tiab] OR thiosulfate[tiab] OR SG1002[tiab])) AND (“2007/01/01” [Date-Publication]: “2025/10/30” [Date-Publication]). Web of Science: TS = (“heart failure” OR “ventricular remodel*” OR “cardiac remodel*” OR “left ventricular hypertrophy” OR “myocardial fibrosis”) AND TS = (“hydrogen sulfide” OR H_2_S OR “cystathionine gamma-lyase” OR CSE OR “cystathionine beta-synthase” OR CBS OR “3-mercaptopyruvate sulfurtransferase” OR 3-MST) AND TS = (“ACE inhibitor*” OR ARB OR “angiotensin receptor blocker*” OR sacubitril OR valsartan OR beta-blocker* OR carvedilol OR metoprolol OR spironolactone OR eplerenone OR SGLT2 OR dapagliflozin OR empagliflozin OR hydralazine OR “isosorbide dinitrate” OR GYY4137 OR NaHS OR thiosulfate OR SG1002); PY = 2007–2025. Scopus: TITLE-ABS-KEY((“heart failure” OR “ventricular remodel*” OR “cardiac remodel*” OR “left ventricular hypertrophy” OR “myocardial fibrosis”) AND (“hydrogen sulfide” OR H_2_S OR “cystathionine gamma-lyase” OR CSE OR “cystathionine beta-synthase” OR CBS OR “3-mercaptopyruvate sulfurtransferase” OR 3-MST) AND (“ACE inhibitor*” OR ARB OR sacubitril OR valsartan OR beta-blocker* OR carvedilol OR metoprolol OR spironolactone OR eplerenone OR SGLT2 OR dapagliflozin OR empagliflozin OR hydralazine OR “isosorbide dinitrate” OR GYY4137 OR NaHS OR thiosulfate OR SG1002)) AND PUBYEAR > 2006 AND PUBYEAR < 2025. Cochrane Library: ([mh “Heart Failure”] OR “heart failure”:ti,ab,kw OR “ventricular remodeling”:ti,ab,kw OR “cardiac remodeling”:ti,ab,kw OR “left ventricular hypertrophy”:ti,ab,kw OR “myocardial fibrosis”:ti,ab,kw) AND ([mh “Hydrogen Sulfide”] OR “hydrogen sulfide”:ti,ab,kw OR H_2_S:ti,ab,kw OR CSE:ti,ab,kw OR CBS:ti,ab,kw OR “3-MST”:ti,ab,kw) AND (“ACE inhibitor”:ti,ab,kw OR ARB:ti,ab,kw OR sacubitril:ti,ab,kw OR beta-blocker:ti,ab,kw OR spironolactone:ti,ab,kw OR eplerenone:ti,ab,kw OR SGLT2:ti,ab,kw OR dapagliflozin:ti,ab,kw OR empagliflozin:ti,ab,kw OR hydralazine:ti,ab,kw OR “isosorbide dinitrate”:ti,ab,kw OR GYY4137:ti,ab,kw OR NaHS:ti,ab,kw OR thiosulfate:ti,ab,kw OR SG1002:ti,ab,kw), publication years 2007–2025.

**Table 3:** SYRCLE risk-of-bias assessment for the 14 unique preclinical studies included in the primary synthesis.

Study	Seq. Generation	Baseline	Allocation Concealment	Random Housing	Personnel Blinding	Outcome Blinding	Incomplete Data	Selective Reporting
Snijder 2015 [28]	Unclear	Low	Unclear	Unclear	Unclear	Unclear	Low	Low
Ahmad 2016 [13]	Unclear	Unclear	Unclear	Unclear	Unclear	Unclear	Unclear	Unclear
Li 2018 [29]	Low	Low	Unclear	Unclear	Unclear	Low	Low	Low
Shao 2017 [30]	Unclear	Unclear	Unclear	Unclear	Unclear	Unclear	Unclear	Unclear
Doiron 2024 [31]	Unclear	Low	Unclear	Unclear	Unclear	Unclear	Low	Unclear
Zhang 2013 [32]	Unclear	Low	Unclear	Unclear	Unclear	Unclear	Low	Unclear
Sun 2015 [33]	Unclear	Unclear	Unclear	Unclear	Unclear	Unclear	Unclear	Unclear
Polhemus 2013 [23]	Unclear	Unclear	Unclear	Unclear	Unclear	Unclear	Unclear	Unclear
Wu 2018 [11]	Unclear	Low	Unclear	Unclear	Unclear	Unclear	Low	Low
Huang 2012 [34]	Unclear	Unclear	Unclear	Unclear	Unclear	Unclear	Unclear	Unclear
Huang 2013 [12]	Unclear	Low	Unclear	Unclear	Unclear	Unclear	Low	Unclear
Kondo 2013 [35]	Unclear	Low	Unclear	Unclear	Unclear	Low	Low	Low
Li 2016 [36]	Unclear	Low	Unclear	Unclear	Unclear	Unclear	Unclear	Unclear
Yu 2017 [37]	Unclear	Low	Unclear	Unclear	Unclear	Unclear	Low	Unclear
Low risk				Unclear risk				

Note. Green = low risk, yellow = unclear risk. Most domains were judged unclear because reporting was insufficient, particularly for sequence generation, allocation concealment, random housing, and blinding. These findings lower confidence in the apparent consistency of favorable H_2_S-donor effects. Domain key: Sequence generation; baseline characteristics; allocation concealment; random housing; blinding of personnel; blinding of outcome assessment; incomplete outcome data; selective reporting.

**Table 4:** Characteristics of the 14 unique studies included in the primary H_2_S–cardiac-remodeling synthesis.

Study	Model/Population	Evidence Tier	Intervention	Follow-Up	Key Remodeling and H_2_S-Related Outcomes
Snijder 2015 [28]	SD rats; Ang II infusion	Remodeling surrogate: hypertensive LVH and fibrosis	Sodium thiosulfate or NaHS	3 weeks	LVH, fibrosis, ANP, oxidative stress, NOx, and H_2_S-related measures
Ahmad 2016 [13]	WKY rats; isoproterenol + caffeine	Remodeling surrogate: LV hypertrophy/hemodynamic stress	NaHS	5 weeks	Hypertrophy indices, collagen, BP, oxidative stress, myocardial H_2_S and CSE
Li 2018 [29]	Murine chronic HF model	Overt HF: RAAS/aldosterone-related cardiorenal dysfunction	H_2_S donor	Chronic protocol	Cardiac function, exercise capacity, renal outcomes, and RAAS-related signaling
Shao 2017 [30]	TAC mice + neonatal cardiomyocytes	Remodeling surrogate: pressure-overload hypertrophy	NaHS ± Nrf2/PI3K inhibition	4 weeks post-TAC	Hypertrophy, Nrf2/HO-1, PI3K/Akt, oxidative stress, and CSE/H_2_S
Doiron 2024 [31]	L-NAME + HFD mice; ZSF1 rats	Overt cardiometabolic HFpEF	Empagliflozin; SG1002; combination	5–8 weeks	Diastolic function, exercise capacity, fibrosis, H_2_S/sulfane sulfur, and 3-NT
Zhang 2013 [32]	Aorta–vena cava shunt rats	Overt volume-overload HF	NaHS	8 weeks	Hemodynamics, cardiac structure, H_2_S-related HO-1 expression, and remodeling
Sun 2015 [33]	SHR vs WKY rats	Remodeling surrogate: hypertension-related collagen remodeling	NaHS	9 weeks	CVF, collagen I/III, TGF-beta/Smad, MMP-13/TIMP-1, and H_2_S
Polhemus 2013 [23]	TAC C57BL/6J mice	Overt pressure-overload HF	DATS	12 weeks	LVEF, dilation, fibrosis, angiogenesis, eNOS/Akt/AMPK, and sulfane sulfur
Wu 2018 [11]	Isoproterenol-induced HF mice	Overt catecholamine-associated HF	ZYZ-803 H_2_S-NO hybrid	4 weeks	LV dysfunction, fibrosis, H_2_S-NO crosstalk, and cGMP signaling
Huang 2012 [34]	Abdominal aortic coarctation rats	Remodeling surrogate: pressure-overload LVH/fibrosis	NaHS ± enalapril	5 weeks	LVW/BW, CSA, fibrosis, hemodynamics, H_2_S, CSE, and Ang II
Huang 2013 [12]	Post-MI HF rats	Overt ischemic HF	Controlled-release S-propargyl-cysteine	4 weeks	Cardiac function, infarct/remodeling outcomes, oxidative stress, and H_2_S-related signaling
Kondo 2013 [35]	TAC mice	Overt pressure-overload HF	NaHS/H_2_S-based intervention	4 weeks post-TAC	Function, hypertrophy, fibrosis, angiogenesis/eNOS, oxidative stress, and apoptosis
Li 2016 [36]	STZ-diabetic rats	Diabetic cardiomyopathy	NaHS	8 weeks post-STZ	Hypertrophy, fibrosis, CHOP/caspase-12, and ER-stress-related outcomes
Yu 2017 [37]	Doxorubicin-treated mice	Toxic dilated cardiomyopathy	NaHS	2 weeks post-DOX	EF/FS, fibrosis, oxidative stress, apoptosis, CK-MB/LDH, and H_2_S-related effects

Abbreviations: AAC, abdominal aortic coarctation; AF, atrial fibrillation; Ang II, angiotensin II; BP, blood pressure; CSA, cardiomyocyte cross-sectional area; CSE, cystathionine-γ-lyase; CVF, collagen volume fraction; DATS, diallyl trisulfide; DCA, dichloroacetate; DOX, doxorubicin; EF/FS, ejection fraction/fractional shortening; ER, endoplasmic reticulum; HF, heart failure; H_2_S, hydrogen sulfide; ISO, isoproterenol; LVH, left ventricular hypertrophy; NaHS, sodium hydrosulfide; TAC, transverse aortic constriction.

**Table 5:** Summary of guideline-directed heart failure drug classes, cardiac remodeling effects, and linkage to hydrogen sulfide (H_2_S) signaling.

Drug Class	Established Cardiac-Remodeling Evidence	Integrated HF + H_2_S + Remodeling Evidence	H_2_S-Pathway Interpretation
RAAS inhibitors (ACEIs, ARBs, ARNI)	Robust reverse remodeling in HFrEF, with reduced LV volumes and fibrosis; major trials did not include H_2_S measurements.	No study assessed HF status, H_2_S biology, and remodeling endpoints together.	RAAS activation suppresses CSE/H_2_S signaling; blockade restores CSE expression and myocardial H_2_S in experimental models. Sulfhydryl ACEIs may increase circulating H_2_S. The mechanistic link is plausible but remains clinically unproven.
β-blockers	Clinically strong reverse-remodeling effect, including improved EF and reduced LV dilation; no direct H_2_S readouts in remodeling trials.	No integrated HF + H_2_S + remodeling study was identified.	Catecholamine stress suppresses H_2_S signaling, while H_2_S donors rescue mitochondrial and oxidative injury in ISO/TAC models. Carvedilol increases myocardial H_2_S in non-HF mice. A possible NO-H_2_S-mitochondrial axis is biologically plausible.
MRAs	Reduce mortality and LV dilation in HFrEF; antifibrotic effects are evident, but H_2_S measures are absent from major trials.	None identified.	Aldosterone promotes oxidative stress, inflammation, and collagen deposition-pathways countered by H_2_S donors. MRAs may preserve endogenous H_2_S signaling, but direct evidence is lacking.
SGLT2 inhibitors	Consistent improvements across the EF spectrum, including congestion, outcomes, and diastolic-function-related phenotypes; direct H_2_S readouts are scarce.	One preclinical HFpEF study tested empagliflozin with or without SG1002.	SGLT2 inhibitors improve diastolic function and fibrosis-related outcomes; adding an H_2_S donor produced greater benefit and higher sulfane sulfur in preclinical HFpEF. This suggests potential synergy, but direct upregulation of the H_2_S pathway by SGLT2 inhibitors has not been proven clinically.
Hydralazine-ISDN	Hemodynamic benefit with some outcome improvement; structural reverse-remodeling effect is modest compared with core GDMT classes.	No study assessed hydralazine/ISDN together with H_2_S signaling and remodeling endpoints.	No established direct interaction with endogenous H_2_S synthesis. The class appears neutral with respect to H_2_S signaling based on currently available evidence.
Direct H_2_S donors	Consistent improvements across HF and remodeling models, including EF, LV dilation, fibrosis, hypertrophy, exercise capacity.	Multiple studies met the integrated HF + H_2_S + remodeling criteria.	Donors restore or augment CSE/H_2_S signaling, activate Nrf2/HO-1 and PI3K/Akt pathways, reduce NOX/ROS, improve mitochondrial stress responses, and suppress TGF-β/Smad-driven fibrosis. This is the strongest evidence for H_2_S-driven reverse remodeling.

Abbreviations: ACEI, angiotensin-converting enzyme inhibitor; ARB, angiotensin receptor blocker; ARNI, angiotensin receptor-neprilysin inhibitor; CSE, cystathionine-γ-lyase; EF, ejection fraction; GDMT, guideline-directed medical therapy; HF, heart failure; HFrEF, heart failure with reduced ejection fraction; ISDN, isosorbide dinitrate; LV, left ventricular; MRA, mineralocorticoid receptor antagonist; NOX/ROS, NADPH oxidase/reactive oxygen species; RAAS, renin–angiotensin–aldosterone system; SGLT2, sodium–glucose cotransporter 2.

## Data Availability

All data used and analyzed during the current study are available from the corresponding author on reasonable request.

## Abbreviations

The following abbreviations are used in this manuscript:

3-MST	3-mercaptopyruvate sulfurtransferase
AAC	Abdominal Aortic Coarctation
ACEI	Angiotensin Converting Enzyme
AF	Atrial Fibrillation
Ang II	Angiotensin II
ANP	Atrial Natriuretic Peptide
ARBs	Angiotensin Receptor Blockers
ARNI	Angiotensin receptor–neprilysin inhibitor
CBS	Cystathionine beta-synthase
CSE	Cystathionine gamma-lyase
EF	Ejection Fraction
GDMT	Guideline-Directed Medical Therapy
HF	Heart Failure
HFpEF	Heart Failure with Preserved Ejection Fraction
HFrEF	Heart Failure with Reduced Ejection Fraction
H_2_S	Hydrogen Sulfide
ISDN	Isosorbide Dinitrate
LVH	Left Ventricular Hypertrophy
MeSH	Medical Subject Headings
MRAs	Mineralocorticoid Receptor Antagonists
NaHS	Sodium Hydrosulfide
Nrf2	Nuclear Factor Erythroid 2-Related Factor 2
PICOS	Population, Intervention, Comparator, Outcomes, and Study Design
PRISMA	Preferred Reporting Items for Systematic Reviews and Meta-Analyses
PROSPERO	Prospectively Registered In The International Prospective Register of Systematic Reviews
RAAS	Renin–angiotensin–aldosterone system
RoB	Risk of Bias
SGLT2	Sodium–glucose cotransporter-2
TAC	Transverse Aortic Constriction
